# The Divergence between Self- and Preceptor-Assessments of Student Performance during Advanced Pharmacy Practice Experiences

**DOI:** 10.3390/pharmacy12030079

**Published:** 2024-05-15

**Authors:** Tonya Brim-Dauterman, Shantanu Rao

**Affiliations:** 1 Department of Experiential Education, College of Pharmacy, The University of Findlay, Findlay, OH 45840, USA; tonya.brim@findlay.edu; 2 Department of Pharmaceutical Sciences, College of Pharmacy, The University of Findlay, Findlay, OH 45840, USA

**Keywords:** APPE, self-evaluation, preceptor, APPE evaluation, metacognition

## Abstract

(1) Objectives: A divergence in self- and preceptor-evaluations of clinical skills has been noted during Advanced Pharmacy Practice Experiences (APPEs). The goal of this study was to determine the domains of overestimation of clinical skills by students during their APPE rotations. (2) Methods: Preceptor-assigned grades for APPE rotations from 2017–2022 were analyzed to identify instances of letter grade B or lower. The self- and preceptor-evaluations of APPE rotation were compared to determine the domains of divergence in evaluation between students and preceptors. (3) Results: Between 2017 and 2022, 305 student APPE rotations were graded as B or lower (~14%) by the preceptors. A statistically significant difference was noted between self- and preceptor-assigned letter grades across all practice settings including ambulatory patient care, community pharmacy, general medicine patient care, hospital/health system pharmacy, and special population patient care APPE rotations. In addition, examining the self- and preceptor evaluation rubric for these rotations revealed a statistically significant overestimation of clinical skills by students in all 9 domains of APPE evaluation. Finally, the divergence in the rating of clinical skills between student- and preceptor evaluation was found to be highest in the domains of planning and follow-up of patient care, disease knowledge, and communication with patients. (4) Conclusions: Students who fail to exhibit exemplary practice readiness during APPEs tend to overestimate their clinical skills in all domains of APPE evaluation. The results from our study support the need for additional avenues to assist in the identification of deficits in student learning before APPEs to increase their self-awareness (metacognition).

## 1. Introduction

In the field of healthcare education, particularly in pharmacy practice, the assessment of student performance is a fundamental component of learning and professional development. Advanced Pharmacy Practice Experiences (APPEs) represent a pivotal phase in a pharmacy student’s journey, providing them with the opportunity to apply their theoretical knowledge in real-world clinical settings. The assessment of student’s performance during these experiences is typically conducted through a dual evaluation process, involving both self-assessment by the students themselves and assessment by their preceptors, who are experienced practitioners and educators in the field.

Metacognition during clinical rotations differs from the traditional classroom, as real-time use of knowledge and application are expected [1]. It has been shown that pharmacy students’ metacognition improves during their APPEs, allowing students to better reflect on their shortcomings and improve their clinical skills [2,3]. Intuitively devising strategies to better the self-refection process has been an area of interest for pharmacy educators [4,5]. However, an intriguing and increasingly prevalent phenomenon has emerged in healthcare education—a divergence between self-assessment and preceptor/faculty evaluation of therapeutic decision-making in didactic [6] and experiential curricular components [3,7]. Several studies also confirm that higher performers tend to be better self-evaluators than lower performers [8,9,10]. This divergence raises significant questions about the accuracy of self-assessments, the factors contributing to them, and their implications for pharmacy education and practice.

The current research, therefore, embarks on an exploration of the disparities between self- and preceptor-assessments to determine the domains of overestimation of clinical skills by students during their APPE rotations. Understanding the disparities between self- and preceptor assessments will lay important groundwork for future studies to examine whether they result from differences in perception, cognitive biases, or other intrinsic factors. Additionally, we seek to uncover the potential implications of this divergence on student learning, self-awareness, and the ultimate goal of producing competent and proficient pharmacy practitioners. 

## 2. Methods

Our institute offers a 0 + 6 pharmacy (PharmD) program to students graduating from high school. Following 2 years of pre-professional foundational courses, students complete 3 additional years of professional pharmacy coursework. During the fourth professional year of the Pharm.D. program, students are required to complete 1440 h of APPE, categorized into nine 4-week rotations. Our program offers a hub site model for experiential education, allowing the possibility of multiple APPE rotations at a health system. After each APPE rotation, students are expected to self-assess their learning and APPE performance for the following sections: (1) professionalism on site (accountability, respect, time management, etc.); (2) professionalism—self-learning and assessment (responding to feedback appropriately, initiating responsibility for patient care, demonstrating lifelong learning habits, etc.); (3) communication with patients; (4) interprofessional communication; (5) drug information knowledge; (6) application of drug information; (7) patient care assessment; (8) planning and follow-up of patient care; and (9) disease knowledge. To assist students with this self-evaluation, an APPE orientation session is scheduled before the start of the fourth professional year, during which students are advised how to accurately self-assess their clinical skills using the self-assessment rubric. An identical assessment rubric for student performance for the above-listed domains is also submitted by the preceptor at the culmination of each APPE rotation. The grading scale for these rubrics is structured as follows: 1-unsatisfactory performance, 2-needs improvement, 3-progressing satisfactorily, and 4-exceeds expectation. The assessment submitted by the preceptor makes up the final grade for the student’s APPE rotation using a letter grade scale of A (assessment score of 3.5 or greater), B (score of 2.5–3.49), or F (failure—less than 2.5).

The self- and preceptor assessment of student performance during APPE rotations over the past 5 years (2017–2022) was accessed through our experiential learning management system, CORE^®^ ELMS (West Warwick, RI, USA). Preceptors in our program include clinical preceptors affiliated with the university at their practice sites and faculty preceptors. Since pair-wise comparison is the foundational requirement for the intended analysis, any rotation with a missing self- or preceptor-standard college APPE rubric was excluded from the present data analysis. For students who received a letter grade of B or lower for their APPE rotation, the self- and preceptor-assigned APPE grades were compared to evaluate differences in the rating of observed skills. The cutoff point of a final letter grade of B helped us focus on students who have opportunities for improvement or low performance during their APPE rotations and to observe their self-perception of APPE performance. The self- and preceptor-assigned final grades for APPE rotations were categorized into the ACPE required practice settings of community pharmacy, ambulatory patient care, hospital/health system pharmacy, and inpatient general medicine patient care. In addition, differences between self- and preceptor-assigned grades were also determined for the elective APPEs and the special population practice settings. Next, differences between the self-assessment of performance during APPE were compared to the preceptor assessment of the student’s skills within all 9 sections listed above. This analysis was again restricted to APPE rotations assigned a final letter grade of B or lower by the preceptors, with the expectation of identifying major themes regarding the difference between self- and preceptor evaluation of student performance. The assessment tool is comprehensive in capturing key objectives for all patient care rotations. Some of the domain areas do not apply to all APPE rotations and, hence, were not evaluated by preceptors and/or students. These areas were excluded from our pair-wise analysis. For example, Section 4 focuses on inter-professional interactions, and depending on the rotation type this domain may not have been assessed.

The collected data for this study was organized within a Microsoft Excel spreadsheet (Redmond, WA, USA). The SPSS V.25 (IBM Corp., Armonk, NY, USA) was utilized to conduct statistical analysis for the present study. The difference between self- and preceptor-assigned grades for APPE rotations was determined through the paired sample *t*-test. The Wilcoxon signed-rank test was utilized to determine differences between self- and preceptor assessment of skills during APPE rotations. The statistical difference was deemed significant at *p* ≤ 0.05. This retrospective study design was reviewed and deemed exempt by the University of Findlay’s Institutional Review Board (#1747).

## 3. Results

In our study, between 2017–2022, a total of 2175 student APPE rotations were included as part of our data analysis. In these years, 305 student APPE rotations (~14%) received a letter grade of B or lower from the preceptors. Amongst all rotation types, the community rotation had the least number of preceptor-assigned letter grade B or lower (n = 18). In contrast, special population patient care rotations (intensive care unit, geriatrics, pediatrics, emergency department, infectious disease, etc.) had the highest number of preceptor-assigned letter grade B or lower over the past 5 years (n = 103). The rest of our analysis was focused on those 305 student APPE rotations that had a letter grade of B or lower from the preceptors between 2017–2022.

### 3.1. Self-Assigned Grades for APPE Rotations

Upon analyzing the data over the past 5 years, students receiving a letter grade of B or lower for their APPEs were found to consistently over-evaluate their skills during APPE rotations (Table 1). For these students, significant differences between self- and preceptor-assigned grades were observed for ambulatory patient care (*p* < 0.001), community pharmacy (*p* = 0.003), general medicine patient care (*p* < 0.001), hospital/health system pharmacy (*p* < 0.001), special population (*p* < 0.001), and elective APPE rotations (*p* < 0.001).

### 3.2. Differences across the Various Domains of APPE Assessment

Next, the potential differences between the self- and preceptor assessment of students’ skills were examined for students who received a letter grade of B or lower for their APPE rotations. As summarized in Table 2, compared to their preceptors, these students were found to overestimate their current measure of skillset in all sections of the assessment report. In descending order, the differences between self- and preceptor evaluation of skills (Z-score) were found to be significant for the following domains: professionalism—self-learning and assessment (−10.4, *p* < 0.001), patient care assessment (−10.1, *p* < 0.001), professionalism on site (−9.5, *p* < 0.001), planning and follow up of patient care (−9.5, *p* < 0.001), application of drug information (−9.3, *p* < 0.001), disease knowledge (−9.1, *p* < 0.001), drug information knowledge (−8.9, *p* < 0.001), communication with the patient (−8.6, *p* < 0.001), and interprofessional communication (−7.7, *p* < 0.001).

**Figure 1:**
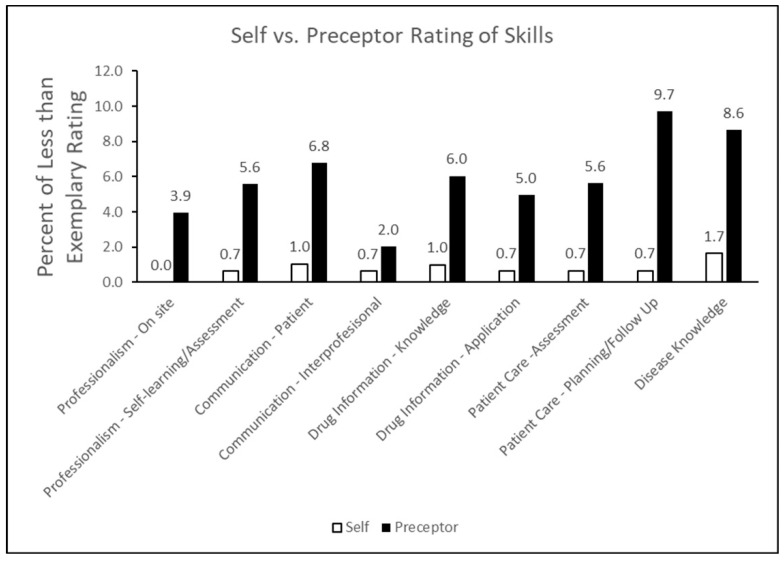
Percent of less-than-exemplary ratings reported by students and preceptors during APPE rotations between 2017–2022.

Finally, for students who were assigned a letter grade of B or lower for their APPE rotations, the percentage of students who self-assessed their skills to be less than satisfactory (unsatisfactory performance or needs improvement) was determined. These values were compared to preceptors’ assessments of students’ skills (Figure 1). The largest deviation in less-than-satisfactory assessment of skills between the self- and preceptor-rating was observed for the planning and follow-up of patient care, followed by disease knowledge and communication with patients. In addition, students overestimated their drug information knowledge (section V) which, combined with their overestimation of disease knowledge, could have impacted their ability to prepare for proper patient care follow-up, as well as appropriate communication with patients, as noted in our analysis. For interprofessional communication, self- and preceptor assessments of students’ skills during APPE rotations were found to be the least different.

## 4. Discussion

The results from our analysis demonstrate that 13.3% of APPE rotations across 5 years received a letter grade of B or lower from preceptors. Amidst these APPE rotations, a statistically significant divergence in self- and preceptor evaluation of clinical skills was found across all 9 sections of the APPE evaluation report. Importantly, for students scoring a letter grade of B or lower during their APPE rotation, the highest degree of error in self-estimation of skills was in the domain of disease knowledge and patient care—planning/follow-up.

The preceptor grades for APPE rotations within our program are submitted using the letter grade format as opposed to the pass/fail system. Pharmacy preceptors’ familiarity and preference for the letter grade system have been reported previously [11]. While direct comparison with preceptor-assigned grades in other pharmacy programs was not part of our analysis, our results were in line with a previous study [12] with regards to the minimal occurrences of APPE grades ‘B or lower’ in community settings as opposed to other patient care rotations. This observation may stem from the fact that students may have had more experience working in the community practice setting during skills lab, internships, 100 h of their required academic IPPE training of 300 h, and the option to earn an additional 100 h in this practice setting as an elective IPPE. Opportunities and expectations within the direct patient care setting are lower during IPPE, thereby not offering adequate exposure to students for self-evaluation of skills in this setting before APPEs [13]. To better prepare students for APPEs, and to bridge the observed divergence between self- and preceptor evaluation of APPE rotations, several approaches can be adopted. Student performance in our existing capstone course can be utilized as a screening tool to identify lapses in students’ knowledge and application of clinical skills [14]. Students exhibiting less than an ideal degree of APPE readiness may be recommended for pre-APPE workshops, as reported in an article recently [15], or other supplemental resources to maximize students’ learning and mentorship during their APPEs.

During APPEs, student schedules are more physically demanding as they relate to accountability during daytime learning. For instance, in the traditional academic setting, students taking 18 credit hours of coursework are expected to be present on campus 72 h per month in comparison to the 160 h per month of work expected while on APPE rotations. This creates a more consistent demand for professionalism, communication, knowledge application from a variety of subject matter, and time management for clinical interventions and projects. A high level of anxiety or burnout from the first three professional years of the pharmacy curriculum has been reported in the literature [16,17,18] and can negatively impact pharmacy students’ acclimatization to the rigors of APPE. A well-directed support system for struggling students can alleviate such concerns and enhance student success during APPEs [19,20].

The three areas of metacognition are knowledge (what concepts, facts, or knowledge is being processed to be used), metacognition control (ability to manage the activity as it relates to the time involved in an item needed to complete the learning or task), and metacognition monitoring (how well the process is being done, whether objectives are being met based on knowledge, and outcomes) [21]. The causes for a letter grade B or lower during APPE rotation vary but can be categorized into professionalism, knowledge, and/or performance. These causes of failure can be related to the 3 major themes of metacognition (knowledge, control, and monitoring), and strategies for improving metacognition have been outlined previously [22]. A variety of other factors can impact student success during APPEs, such as acute social or family situations impacting the ability to concentrate, chronic concerns in managing mental health, lack of curiosity and general desire, language barriers or communication gaps, physical health, inability to prioritize, tardiness, major knowledge gaps, and the ability to apply knowledge to daily clinical functions.

Our observations underscore the need to ensure that students are accurately aware of their current level of clinical, professional, and operational skills. The results from our study support the need for additional avenues to assist in the identification of deficits in student learning before APPEs to increase their self-awareness (metacognition). In doing so, we can expect improvement in the identified areas of deficit revealed in the present analysis. Our future goal is to identify students earlier in the curriculum and assist them in improving their metacognition in a clinical setting. Helping students with personalized monitoring, knowledge checks, and proactive academic advising is envisioned to set them up for meaningful clinical experiences during their APPEs.

The present study has a few limitations. First, the data is from a single pharmacy program and our analysis does not allow the ability to measure subjective differences between preceptors using an objective assessment tool. Also, our analysis does not track individual student performance during APPEs over their final year in the program. Hence, there is a lack of conclusive evidence to suggest personal growth and improvement in metacognition over the last year of the professional program.

In conclusion, our study demonstrates the divergence between self- and preceptor evaluation for students who have less-than-excellent performance during their APPEs. Specifically, for the first time, students’ overestimation of their clinical skills, particularly in the realm of disease knowledge and patient care—planning/follow-up, was revealed in our data analysis.

## Figures and Tables

**Table 1:** Variance between self- and preceptor-assigned letter grades for APPE rotations between 2017–2022.

Category	Mean	SEM	N	*p* Value (Two-Sided)
Ambulatory_GradeB_Self	3.62	0.07	24	<0.001
Ambulatory_GradeB_Preceptor	3.16	0.04	24	
Community_GradeB_Self	3.48	0.10	18	0.003
Community_GradeB_Preceptor	3.10	0.05	18	
GenMed_GradeB_Self	3.42	0.05	46	<0.001
GenMed_GradeB_Preceptor	2.99	0.05	46	
HospitalHealth_GradeB_Self	3.44	0.07	24	<0.001
HospitalHealth_GradeB_Preceptor	3.12	0.04	24	
Special_Popultn_GradeB_Self	3.48	0.03	103	<0.001
Special_Popultn_GradeB_Preceptor	3.09	0.03	103	
Elective_GradeB_Self	3.51	0.03	89	<0.001
Elective_GradeB_Preceptor	3.14	0.02	89	

**Table 2:** Differences in self- and preceptor evaluation of clinical skills in various learning domains assessed during APPE rotations.

Learning Domain	N	Mean	Std. Dev.	Min.	Max.	Z Score	*p*-Value
Professionalism, on-site—Self	305	3.76	0.43	3	4	−9.5	<0.001
Professionalism, on-site—Preceptor		3.35	0.58	1	4		
Professionalism, self-learning and assessment—Self	304	3.65	0.49	2	4	−10.4	<0.001
Professionalism, self-learning and assessment—Preceptor		3.20	0.54	1	4		
Communication with patients—Self	275	3.53	0.52	2	4	−8.6	<0.001
Communication with patients—Preceptor		3.13	0.52	1	4		
Interprofessional communication—Self	197	3.52	0.47	2	4	−7.7	<0.001
Interprofessional communication—Preceptor		3.14	0.39	2	4		
Drug information knowledge—Self	294	3.40	0.51	2	4	−8.9	<0.001
Drug information knowledge—Preceptor		3.04	0.44	1	4		
Application of drug information—Self	300	3.36	0.47	2	4	−9.3	<0.001
Application of drug information—Preceptor		3.01	0.36	1.5	4		
Patient care assessment—Self	301	3.41	0.49	2	4	−10.1	<0.001
Patient care assessment—Preceptor		3.00	0.36	1.5	4		
Planning and follow-up of patient care—Self	296	3.39	0.50	2	4	−9.5	<0.001
Planning and follow-up of patient care—Preceptor		2.99	0.46	1	4		
Disease knowledge—Self	300	3.29	0.49	2	4	−9.0	<0.001
Disease knowledge—Preceptor		2.96	0.36	2	4		

## Data Availability

The raw, de-identified data supporting the conclusions of this article will be made available by the authors on appropriate request.

## References

[1] Rivers M.L., Dunlosky J., Persky A.M. (2020). Measuring Metacognitive Knowledge, Monitoring, and Control in the Pharmacy Classroom and Experiential Settings. Am. J. Pharm. Educ..

[2] Isaacs A.N., Steuber T.D., Howard M.L., Dy-Boarman E.A., Nisly S.A. (2022). Evaluating the Impact of Advanced Pharmacy Practice Experiences on Student Pharmacist Metacognition. Am. J. Pharm. Educ..

[3] Nisly S.A., Sebaaly J., Fillius A.G., Haltom W.R., Dinkins M.M. (2020). Changes in Pharmacy Students’ Metacognition through Self-Evaluation during Advanced Pharmacy Practice Experiences. Am. J. Pharm. Educ..

[4] Briceland L.L., Tackes C.C., Veselov M. (2022). A structured self-reflection approach to improve reflection quality and assessment of advanced pharmacy practice experience professionalization. J. Am. Coll. Clin. Pharm..

[5] Fierke K.K., Lepp G.A., Maxwell W.D., Hager K.D., Sucher B.J. (2019). Improving advanced pharmacy practice experiences with an intention/reflection practice. Curr. Pharm. Teach. Learn..

[6] Abeyaratne C., Nhu T., Malone D. (2022). Self-Assessment of Therapeutic Decision-Making Skills in Pharmacy Students. Am. J. Pharm. Educ..

[7] Helmer A.M., Slater N.A., Marlowe K.F., Surry D.W., Mccoy E.K. (2020). Comparing faculty evaluations of student journal club presentations with student self- and peer evaluations during advanced pharmacy practice experiences. Curr. Pharm. Teach. Learn..

[8] Hacker D.J., Bol L., Horgan D.D., Rakow E.A. (2000). Test prediction and performance in a classroom context. J. Educ. Psychol..

[9] Hartwig M.K., Was C.A., Isaacson R.M., Dunlosky J. (2012). General knowledge monitoring as a predictor of in-class exam performance. Br. J. Educ. Psychol..

[10] Austin Z., Gregory P.A. (2007). Evaluating the accuracy of pharmacy students’ self-assessment skills. Am. J. Pharm. Educ..

[11] Varner L.H., Radhakrishnan R., Rollins B.L. (2018). Preceptor’s grading scale preference for student pharmacy practice experience and assessment of the common grading scale among US schools of pharmacy. Curr. Pharm. Teach. Learn..

[12] Tofade T., Shepler B.M., Feudo D.M., Kieser M.A., Jolowsky C., Miller M.L., Sullivan D., Vos S.S., Brueckl M., Walker P.C. (2018). Grading trends and evaluation of student performance across advanced pharmacy practice experiences (APPE) in the Big Ten Academic Alliance (The GRAPPES study). Curr. Pharm. Teach. Learn..

[13] Maes M.L., Barnett S.G., Porter A.L. (2024). A Call to Action for Integrating Introductory Pharmacy Practice Experiences with Purpose. Am. J. Pharm. Educ..

[14] Minshew L.M., Yi J., Morbitzer K.A., McLaughlin J.E. (2020). Use of Capstone Experiences in Pharmacy Education to Synthesize and Apply Students’ Knowledge and Skills. Am. J. Pharm. Educ..

[15] Alexander K., Eiland L.S., Andrus M. (2022). Teaching of the patient workup process improves students’ perceptions of preparation for advanced pharmacy practice experiences. Curr. Pharm. Teach. Learn..

[16] Jacoby J.L., Cole J.D., Ruble M.J., Smith A.B., Laubach L.T., Greenberg M.R., Macfarlan J.E., DeWaay D.J., Barraco R.D., Shigo E. (2021). Measures of Burnout and Empathy in United States Doctor of Pharmacy Students: Time for a Change?. J. Pharm. Pract..

[17] Votta R.J., Benau E.M. (2014). Sources of stress for pharmacy students in a nationwide sample. Curr. Pharm. Teach. Learn..

[18] Zeeman J.M., Benksy H.P., Minshew L.M. (2023). Pharmacy Student Stress and Time Use in Pre-Clinical and Clinical Students. Am. J. Pharm. Educ..

[19] Cat T.B., El-Ibiary S.Y., Lee K.C. (2023). Evaluation of a Well-being Promotion (WelPro) Program on Advanced Pharmacy Practice Experience (APPE) Student Burnout. Am. J. Pharm. Educ..

[20] Truhlar L.M., Durand C., Cooper M.R., Goldsmith C.W. (2022). Exploring the effects of a smartphone-based meditation app on stress, mindfulness, well-being, and resilience in pharmacy students. Am. J. Health Syst. Pharm..

[21] Dunlosky J., Metcalfe J. (2009). Metacognition.

[22] Medina M.S., Castleberry A.N., Persky A.M. (2017). Strategies for Improving Learner Metacognition in Health Professional Education. Am. J. Pharm. Educ..

